# Wildfire impact on soil microbiome life history traits and roles in ecosystem carbon cycling

**DOI:** 10.1093/ismeco/ycae108

**Published:** 2024-08-27

**Authors:** Amelia R Nelson, Charles C Rhoades, Timothy S Fegel, Holly K Roth, Marcos V Caiafa, Sydney I Glassman, Thomas Borch, Michael J Wilkins

**Affiliations:** Department of Soil and Crop Sciences, Colorado State University, Fort Collins, CO 80523, United States; Rocky Mountain Research Station, United States Forest Service, Fort Collins, CO 80526, United States; Rocky Mountain Research Station, United States Forest Service, Fort Collins, CO 80526, United States; Department of Soil and Crop Sciences, Colorado State University, Fort Collins, CO 80523, United States; Department of Microbiology and Plant Pathology, University of California Riverside, Riverside, CA 92521, United States; Department of Microbiology and Plant Pathology, University of California Riverside, Riverside, CA 92521, United States; Department of Soil and Crop Sciences, Colorado State University, Fort Collins, CO 80523, United States; Department of Chemistry, Colorado State University, Fort Collins, CO 80523, United States; Department of Soil and Crop Sciences, Colorado State University, Fort Collins, CO 80523, United States

**Keywords:** soil microbiome, metagenomics, wildfire, traits

## Abstract

Wildfires, which are increasing in frequency and severity with climate change, reduce soil microbial biomass and alter microbial community composition and function. The soil microbiome plays a vital role in carbon (C) and nitrogen (N) cycling, but its complexity makes it challenging to predict post-wildfire soil microbial dynamics and resulting impacts on ecosystem biogeochemistry. The application of biogeochemically relevant conceptual trait-based frameworks to the soil microbiome can distill this complexity, enabling enhanced predictability of soil microbiome recovery following wildfire and subsequent impacts to biogeochemical cycles. Conceptual frameworks that have direct links to soil C and N cycling have been developed for the soil microbiome; the Y-A-S framework overviews soil microbiome life history strategies that have tradeoffs with one another and others have proposed frameworks specific to wildfire. Here, we aimed to delineate post-wildfire changes of bacterial traits in western US coniferous forests to inform how severe wildfire influences soil microbiome recovery and resultant biogeochemical cycling. We utilized a comprehensive metagenome-assembled genome catalog from post-wildfire soils representing 1 to 11 years following low- and high-severity burning to identify traits that enable the persistence of microbial taxa in burned soils and influence ecosystem C and N cycling. We found that high-severity wildfire initially selects for fast growers and, up to a decade post-fire, taxa that invest in genes for acquiring diverse resources from the external environment, which in combination could increase soil C losses. This work begins to disentangle how climate change–induced shifts in wildfire behavior might alter microbially mediated soil biogeochemical cycling.

## Introduction

The soil microbiome plays critical roles in myriad ecosystem services, including the fostering of plant productivity [1] and regulating soil C transformation and storage [2, 3]. These ecosystem services can be impacted through a variety of different disturbance types, with wildfire representing a disturbance with both short- and long-term effects on microbial composition and function [4–7]. Recent insights into post-disturbance soil microbiome dynamics have leveraged multi-omic (e.g. metagenomics, metabolomics) [6], pyrocosm [8, 9], or laboratory [10] approaches, but the diversity and heterogeneity of the soil microbiome can pose challenges when interpreting the scalability of these findings across different ecosystems and wildfires, along with their cascading impacts on ecosystem processes (e.g. C and N fluxes).

In plant communities, ecologists have addressed these challenges by developing conceptual life history strategy frameworks to summarize key plant fitness traits that govern post-disturbance successional trajectories. One such framework, Grimes’ C-S-R [11], categorizes plants through three primary life history strategies: competitors (C), stress tolerators (S), and ruderal (R) species, with tradeoffs between traits that represent each strategy. Competitors excel at maximizing resource capture in productive or undisturbed systems (e.g. perennials), stress tolerators are associated with continuously low resource conditions (e.g. cacti), and ruderal species are successful in recently disturbed systems and have traits like shade intolerance, fast growth, or asexual reproduction. These three strategies have been shown to apply broadly across ecosystems, adequately represent and distill the taxonomic diversity in plant communities [12], and help predict post-disturbance plant community compositional shifts [13, 14].

The success of conceptual life history strategy frameworks in plant ecology has led microbial ecologists to adapt these ideas for the complex soil microbiome; a microbe that is classified as a competitor might have a large genome and high catabolic diversity, a stress tolerator microbe is proposed to encode stress tolerant genes or grow slowly, and a ruderal microbial species might be a spore former that can grow quickly [15]. Malik *et al*. [16] further proposed a new framework, the Y-A-S framework, as a rendition of Grimes’ C-S-R specifically modified to represent the soil microbiome. Here, the three microbial life history strategies are suggested to be high yield (Y), or taxa that maximize growth efficiency and thrive in resource-abundant conditions, resource acquisition (A), taxa that primarily invest in resource acquisition machinery (e.g. extracellular enzymes) and thrive in conditions where resources are complex or depleted, and stress tolerators (S) that invest energy into mechanisms for persisting during fluctuating soil conditions (e.g. osmolyte production, desiccation). Importantly, all three life history strategies are proposed to be linked to emergent C cycling, making this approach valuable for predicting disturbance impacts to ecosystem biogeochemistry.

There are also conceptual frameworks proposed for specific disturbances. One such example is wildfire, which is a unique soil disturbance as it causes distinct pulse and press disturbance impacts that differ with fire severity [6, 7, 17]. The initial pulse disturbance of a severe wildfire is associated with combustion of surface soil and aboveground vegetation, while the longer-term press disturbance is defined by lasting impacts to aboveground vegetation structure and altered soil chemistry (e.g. increased pH, changes in soil C quality and quantity) [4, 18, 19]. Severe wildfire depletes soil microbial biomass [20] and diversity [6, 7], alters microbially mediated C and N functionality [4–6], and selects for putative pyrophilous bacterial taxa (e.g. Actinobacteria *Arthrobacter* and Proteobacteria *Massilia*) that exhibit specific traits to persist in post-wildfire soils. To help explain the drivers of post-wildfire soil microbial succession, recent studies [21, 22] have proposed fire microbial ecology frameworks that include pyrophilous life history traits that are advantageous for soil microbes during and following fire. These traits include heat resistance, ability to degrade fire-transformed soil C, fast growth, and ability to withstand high soil pH induced by burning. Again, these traits have clear links to ecosystem biogeochemistry. For example, copiotrophic taxa are hypothesized to grow less efficiently, causing increased soil C loss via respiration [23]. Therefore, applying conceptual life history strategy frameworks to the post-wildfire soil microbiome might help distill complexity and disentangle the feedbacks between wildfire behavior, the soil microbiome, and ecosystem biogeochemistry.

Here, we use genome-resolved metagenomics to assess the impact of severe wildfire on life history strategies encoded in the soil microbiome and predict the subsequent impact on microbially mediated ecosystem biogeochemistry. We generated a comprehensive soil metagenome-assembled genome (MAG) database from soils collected across an 11-year post-fire chronosequence in coniferous forests (CO, USA) burned at low and high severity, along with unburned controls. The use of MAGs provides insights into the function of bacterial taxa enriched in a prior amplicon sequencing study on this sample set [7] and found across multiple post-fire ecosystems (e.g. boreal forest [24], California chaparral [25], redwood tanoak forest [21]); function can be challenging to predict from amplicon sequencing alone, especially on environmental samples where many taxa do not yet have cultured isolates [26]. Furthermore, although traits and functions can be analyzed using gene-resolved metagenomics, this approach limits the linkages that can be made between function and taxonomy, and prevents the interpretation of genes in a genomic context [27]. In comparison, genome-resolved analyses provide opportunities to assess the genomic functional potential for each community member within a community; e.g. prior work by our group [6] demonstrated the functional potential for degradation of pyrogenic organic matter post-fire, but noted that incomplete pathways in the majority of genomes suggested cross-feeding of degradation intermediates between community members. Using a similar approach in this study, we hypothesized that life history strategy profiles would vary with burn severity and that fire would select for previously identified pyrophilous taxa with stress tolerance traits (e.g. persisting during higher heat, decreased soil moisture) and that over time these taxa would be replaced with those that invest in resource acquisition traits due to the complexity of fire-transformed soil C. Furthermore, we hypothesized that wildfire-induced shifts in microbial life history strategy traits might lead to enhanced soil C losses in a severely burned system over 11 years post-fire.

## Materials and methods

### Field campaign and sample set

Soil samples were collected in 29 and 30 July 2021 across a chronosequence of wildfire burn scars in northern CO (USA). Wildfire burn scars sampled included the 2010 Church’s Park fire (representing 11 years post-fire), 2016 Beaver Creek fire (5 years post-fire), 2018 Ryan fire (3 years post-fire), and the 2020 Mullen fire (1 year post-fire) (Fig. S1). At each burn scar, mineral soil samples were collected from the 0–5 cm depth and manually homogenized from three separate transects that included both low- and high-severity-impacted soils, along with an unburnt control. Burn severities were determined using US Forest Service guidelines [28]; low and high severity sites had >85% and <20% surficial organic matter, respectively, which we quantified visually within each 1-m^2^ sampling plot. See Caiafa *et al*. [7] for more details on the sampling campaign. Along with the aforementioned samples, we also used metagenomes from shallow soil samples described in Nelson *et al*. [6], which included Ryan fire sites sampled 1 year following the fire in 2019. The entire sample set utilized here includes 42 total samples representing both low- and high-severity-impacted soils up to 11 years following burning (Table S1). Sample metadata are included in Supplementary Data.

### Soil chemistry and microbial biomass

We evaluated soil properties and microbial biomass on a subset of samples to gauge changes across the chronosequence. To analyze inorganic forms of soil N (NO_3_–N and NH_4_–N), samples were passed through a 4-mm-mesh sieve and extracted with 2 M KCl. Extracts were analyzed for NO_3_–N and NH_4_–N by colorimetric spectrophotometry [29] (Lachat Company, Loveland, CO, USA). We analyzed the NO_3_–N and NH_4_–N and dissolved organic C (DOC) and total dissolved N (TDN) released during warm water extracts [30] using ion chromatography (NH_4_–N and NO_3_–N; Thermo-Fisher Corporation, Waltham, MA, USA) and TOC-VCPN total organic carbon analyzer (DOC and TDN; Shimadzu Corporation, Columbia, MD, USA). Soil pH was analyzed in a 1:1 soil to deionized water slurry after 1 h of agitation [31] using a temperature-corrected glass electrode (Hach Scientific, Loveland, CO, USA). A subset of 27 samples were sent to the Soil Health Assessment Center at the University of Missouri for phospholipid fatty acid (PLFA) and neutral lipid fatty acid analysis using the Buyer and Sasser [32] extraction method with a Bligh–Dyer extraction [33] and MIDI Sherlock software (v6.3; MIDI, Inc., Newark, DE, USA). All data are included in Supplementary Data.

### DNA extractions and metagenomic sequencing processing

Total DNA was extracted from bulk soil (*n* = 36) using the QIAGEN DNeasy PowerSoil Pro (QIAGEN, Germantown, MD, USA) kit following the manufacturer’s protocol. Metagenomic sequencing libraries were prepared using the KAPA HyperPrep library preparation kit and samples were sequenced at the Joint Genome Institute using the Illumina NovaSeq S4 platform (2 × 151 bp). Sequencing depth ranged from 10 to 93 Gbp (Supplementary Data). Sequencing adapter sequences were removed from raw reads using BBduk (https://jgi.doe.gov/data-and-tools/bbtools/bb-tools-user-guide/bbduk-guide/) and reads were trimmed with Sickle [34] (v1.33). For each sample, trimmed reads were assembled into contiguous sequences (contigs) using the *de novo* de Bruijn assembler MEGAHIT v1.2.9 using kmers [35] (minimum kmer of 27, maximum kmer of 127 with step of 10). Assembled contigs (>2500 bp) were binned using MetaBAT [36] with default parameters (v2.12.1). MAG quality was estimated using checkM [37] (v1.1.2) and taxonomy was assigned using GTDB-Tk [38] (v2.1.1). MAGs from these metagenomes were dereplicated using dRep [39] (default parameters, v3.0.0) and low-quality MAGs (<50% completion and >10% contamination) [40] were removed, resulting in 228 medium- and high-quality MAGs. The final 228 dereplicated medium- and high-quality MAGs from this sequencing effort were combined with MAGs presented in Nelson *et al*. [6], and this dataset was dereplicated (using dRep) to create the final nonredundant Fire-Responding Ecogenomic database (FiRE-db) v1.0. This resulted in 825 final medium- and high-quality MAGs within the database (620 from Nelson *et al*. [6], 205 from new sequencing campaign; Supplementary Data). Because of the large range of sample sequencing depth, reads >15 Gbp were rarefied to 15 Gbp for mapping and estimating coverage of MAGs across samples. Rarefied reads were mapped to MAGs using Bowtie2 [41] (v2.3.5) and MAG coverage across samples was calculated using coverM genome (v0.6.0) with the “Trimmed Mean” (hereafter referred to as TMM) method.

To identify traits encoded within the genomes, *microTrait* [42] was run using R v4.1.2 and default settings and rules. Briefly, *microTrait* uses a library of Hidden Markov Models (HMMs; *microTrait*-HMM) for protein detection in MAGs and then maps the presence/absence of different proteins to traits using logical rules (e.g. for NO_2_^−^ reduction to be present in a MAG, need *nirS* or *nirK* present; *microTrait*-rules). Traits that are profiled using *microTrait* fall into three categories: resource acquisition, resource use (energy generating), and stress tolerance traits. Resource acquisition traits include those that facilitate substrate uptake (e.g. specialized membrane transporters or exoenzymes), resource use includes energy metabolism traits and genes that catalyze redox reactions, and stress tolerance traits in *microTrait* are those that facilitate microbial persistence during conditions that may adversely affect microbial growth or survival (e.g. molecular chaperones, heat shock, desiccative stress). See Supplementary Data, Sheet E for all traits profiled across MAGs. To further identify putative pyrogenic C (PyC) degraders, we used HMMER [43] against Kofamscan HMMs [44] and HMMs from the CANT-HYD [45] database. Maximum genome doubling times of genomes were estimated via codon usage bias using gRodon [46] (v2.0.0). We used a threshold of 5 h to delineate putative fast and slow growers because, when using gRodon, there is no longer a signal of growth optimization via codon usage bias (CUB) with estimated growth rates >5 h [46]. A machine learning model was used to estimate pH preference of MAGs based on profiles of 56 genes encoded within the MAGs [47]. To predict the subcellular localization of the protein sequences of CAZymes with substrate specificity encoded within the dominant MAGs, we subset the annotated CAZymes to those included in Piton *et al*. [48] and used PSORTb (v3.0.3; [49]) with default settings, retaining localization sites with confidence score >7.5.

To focus on MAGs most representative of the different treatments, we subset the FiRE-db to the top 50 most abundant MAGs (via coverage) in each treatment (e.g. 3-year post-fire high severity) that resulted in >80% read recruitment for all samples, with an exception of Ryan fire high severity (65% read recruitment). This is likely due to the large number of MAGs from samples collected from the Ryan fire. The range of coverages of dominant MAGs for each condition ranged from an average of 0.845 to 23.500 (Table S2) with variability between treatments introduced by variable quality of metagenomic sequencing, assembly, or successful binning from the metagenome. The MAG list is included in Supplementary Data.

### Microbial community analyses and statistics

To identify how the soil microbiome compositional and traits characterized via genome-resolved metagenomic sequencing differed by fire treatment (unburned, low- and high-severity wildfire) and time post-fire (1, 3, 5, and 11 years), statistical analyses were performed using R (v4.3.0) [50] with significance values accepted at *P* <.05 across all tests. To visualize compositional differences in bacterial community composition across fire treatments and time post-fire, non-metric multidimensional scaling (NMDS) was conducted using Bray–Curtis dissimilarity in the vegan package [51] with ellipses drawn at 95% confidence intervals. The number of *microTrait* assigned Y, A, and S traits within MAGs was correlated with the resulting NMDS space using the “envfit” function in the vegan package with a Bonferroni *P*-value correction for multiple tests. To statistically test the compositional differences, nonparametric permutational multivariate analyses of difference [52] were performed again using Bray–Curtis dissimilarity matrices with the “adonis2” function in vegan [51]. Differences in soil chemistry variables, PLFA, MAG # of encoded A traits, MAG size, MAG pH preference, among other variables (explicitly mentioned in figure captions if test was used) were assessed and tested using pairwise Wilcoxon signed-rank tests with a Bonferroni *P*-value adjustment for multiple tests using the function “stat_compare_means” in the package ggpubr [53]. Further testing for interactive effects of fire treatment and time post-fire was tested using the function “aov” in the car package [54]. Spearman rho tests were used to test the strength of correlation between the number of encoded MAG aromatic degradation genes and the MAG coverage within different conditions using the “cor.test” function within the stats core R package [50]. The R package ggplot2 [55] was used to make all plots, which were all edited and made more clear for readers (e.g. excess axis legends removed) in Adobe Illustrator (v28.3).

## Results and discussion

### MAG catalog representing diverse soil bacterial taxa across time following wildfire

To characterize how wildfire influences soil microbiome life history strategy traits related to ecosystem C and N cycling, we performed shotgun metagenomic sequencing on 36 surface (0–5 cm depth) soil samples collected across a 1–11-year post-wildfire chronosequence in northern CO and southern WY, and included varying burn severities (low and high severity) and unburned control soils (Fig. S1; Table S1). Through the sequencing of DNA extracted from these samples (~2.3 Tb), we generated a unique catalog of fire-responding MAGs; 228 MAGs reconstructed from this sequencing effort were combined with a MAG catalog (*n* = 637 MAGs) from a study conducted 1 year post-fire in the Ryan fire [6] to generate the Fire-Responding Ecogenomic database (FiRE-db) v1.0. This unique resource encompassed 825 dereplicated, medium- and high-quality MAGs, including MAGs representing the bacterial phyla Actinobacteria (*n* = 311), Proteobacteria (*n* = 212), and Bacteroidota (*n* = 73) along with putative pyrophilous taxa identified in previous post-wildfire studies [6, 24, 25] (*Arthrobacter*, *n* = 12; *Blastococcus*, *n* = 9; Massilia, *n* = 9) (Fig. S2). This comprehensive collection of MAGs within FiRE-db was used to investigate the impact of wildfire on soil microbiome functional profiles and life history strategies across both time (1–11 years post-fire) and disturbance severity (control, low and high severity) in the Colorado Rockies to characterize how severe wildfire could influence microbially mediated ecosystem C and N cycling.

### Wildfire drives soil microbiome community composition

**Figure 1:**
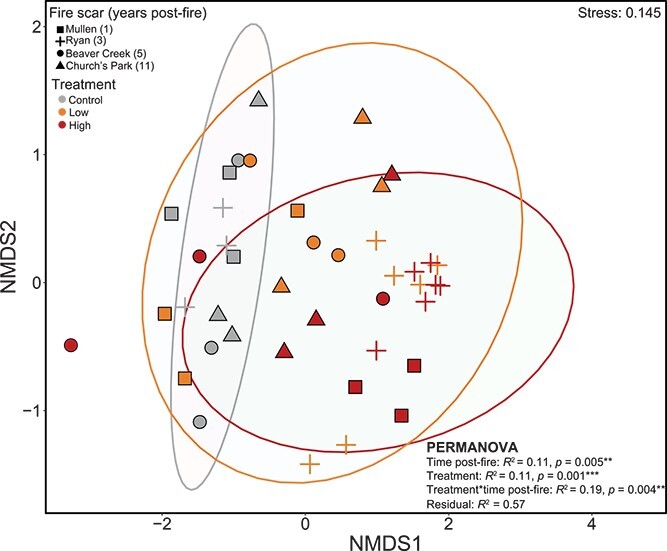
Non-metric multidimensional scaling (NMDS) ordination of Bray–Curtis dissimilarity between samples based on MAG relative abundance profiles. Ellipses show 95% confidence intervals around each treatment.

**Figure 2:**
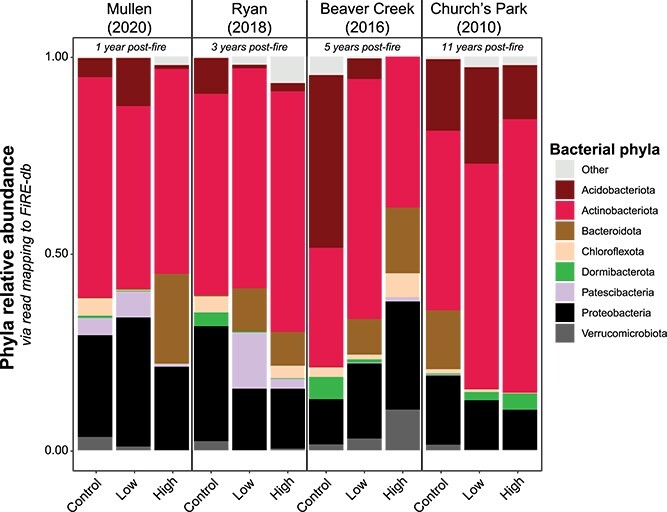
Relative abundance of bacteria phyla across treatments, averaged by triplicate, estimated by mapping metagenomic reads to FiRE-db.

Wildfire drove long-term (11 year) declines in microbial biomass in burned surface soils, with greater impacts in high-severity soils (average biomass loss of 43.6% and 62.3% with low- and high-severity fire, respectively) and negligible recovery with time (biomass loss with high severity −64.9% 1 year post-fire to −67.2% 11 years post-fire; Fig. S3). There were also evident wildfire-driven shifts in bacterial community composition. Bacterial compositional shifts inferred from metagenomic read mapping to the MAG catalog confirmed prior broad compositional trends found via 16S rRNA gene sequencing [7], with time post-fire (explaining ~10.82% of the variation) and burn treatment (11.48%) significantly driving community composition (Fig. 1, Table S3), along with significant effects of the interaction of time post-fire and burn treatment (19.7%; Fig. 1, Table S2). The effect of treatment varied depending on the time post-fire, with the effect of fire treatment (i.e. control, low severity, high severity) waning with time following wildfire (Table S4). Further mirroring the corresponding marker gene data [7], MAG coverage profiles generally revealed a high-severity-fire-induced reduction of Verrucomicrobia and Acidobacteria accompanied by an increase in Actinobacteria (Fig. 2) that was most evident in the more recently burned high-severity-impacted soils.

### Signature of wildfire on soil microbiome life history strategies

The impact of wildfire on the distribution of life history traits across the soil microbiome was investigated using *microTrait* [42], a tool that uses genes and predicted metabolic pathways to infer microbial processes that are ecologically relevant for soil C and N cycling [16]. The identified traits can be associated with three broad categories, resource acquisition (A), resource use (energy generating; Y), or stress tolerance (S), known broadly as the Y-A-S conceptual framework [16]. Y-A-S was proposed as an alternative to Grime’s C-S-R framework for plant communities [56] to summarize the three key life history strategies of soil microbes [16]. Y-Strategists allocate resource use to cellular growth by investing in assimilatory pathways; A-strategists invest into acquiring resources via production of extracellular enzymes or specialized membrane transporters; and S-strategists invest energy into stress tolerance processes aiding in biomolecular damage repair, desiccation protection, thermal resistance, or osmolyte production. This framework has been used to characterize soil microbiomes across ecosystem types [57–60] and has been previously inferred from MAG data [48, 61].

We selected the top 50 most abundant MAGs (via coverage) within each treatment (e.g. 1 year post-fire high severity), which generally represented at least 80% of total metagenome coverage (Supplementary Data). This subset retained 320 MAGs and preserved many of the compositional shifts seen in the entire MAG catalog and coupled 16S rRNA gene sequencing dataset [7] (e.g. loss of Acidobacteria and Verrucomicrobia from unburned to high-severity-fire-impacted soils; Fig. S4, Fig. 2). There was overlap between the dominant MAGs across treatments (Fig. S5a), with more shared dominant MAGs between high-severity-impacted sites that were closer in time (Fig. S5b). This trend was absent in low-severity-impacted sites, revealing the stronger selective pressure of high-severity wildfire that wanes with time.

**Figure 3:**
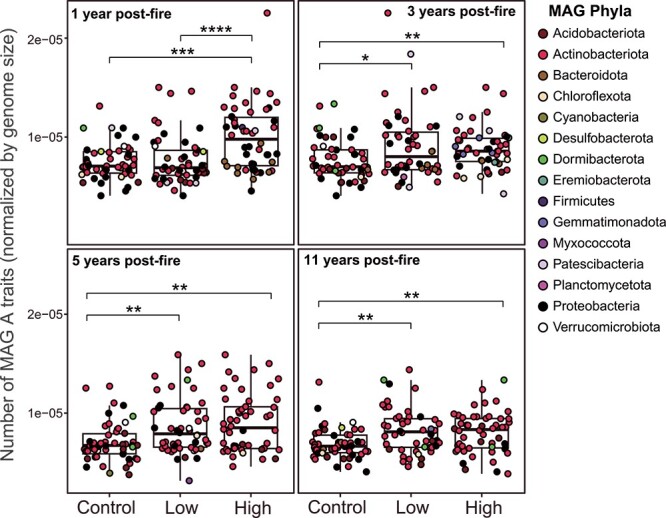
Total number of *microTrait* annotated resource acquisition (A) traits normalized by MAG size (bp) in the top 50 dominant MAGs across all conditions. The lower and upper hinges of the boxplots represent the 25th and 75th percentiles, respectively, and the middle line is the median. The whiskers extend from the median by 1.5× the interquartile range. Points represent individual MAGs. Significant differences between conditions indicated with asterisks as indicated by Wilcoxon rank-sum test. ^*^P < .05, ^*^^*^P < .01, ^*^^*^^*^P < .001.

Soils impacted by high-severity wildfire were selected for taxa with greater genomic investment into A strategy traits than control and low-severity-impacted soils over the entire 11-year chronosequence (Fig. 3). Factorial analysis of variance (ANOVA) results showed that treatment and time post-fire had a significant effect on the number of encoded A strategy traits with a significant interaction between treatment and time post-fire indicating that the impact of treatment varied over time (Table S5). This pattern was largely absent for Y strategy traits (Fig. S6; Table S5). S Strategy traits were enriched in later years [5 and 11] post-fire, suggesting that these are not enriched by the initial pulse disturbance of wildfire (e.g. heat), but instead by the longer-term impacts such as altered soil chemistry or lack of vegetation reestablishment in these sites (54; Fig. S6; Table S5). The increased dominance of A-strategists up to 11 years post-high-severity wildfire could be caused by fire-induced resource scarcity and lack of forest recovery even at the Church’s Park fire sites (11 years post-fire) or resource complexity, resulting in the selection for taxa that increase investment into resource capture [16]. Increased A traits included carbohydrate-active enzymes (CAZymes), with an increase in encoded CAZymes in high-severity soils 1 year post-fire, and variability in the enrichment of different CAZymes over the chronosequence (Fig. S7). In the immediate aftermath of fire (1 year), the active recruitment of resources from the external environment was highlighted by the enrichment of CAZymes likely localized to the cellular periplasm or extracellular space (via PSORTb [49]; Fig. S7). Increased microbial investment into A strategy traits may lead to greater soil C loss up to a decade following wildfire due to hypothesized inefficient cellular growth (i.e. lower carbon use efficiency, CUE) [62].

Genes encoding for aminopeptidases and metalloendopeptidases, enzymes that cleave bonds in proteins and peptides, were also enriched in the high-severity-impacted soil microbiome (Fig. S8). Microbial necromass has been hypothesized to increase following burning [6, 22] and the enrichment of aminopeptidases suggests that necromass represents a key resource supporting microbial activity following wildfire. Peptidases might also serve a critical function in post-wildfire soils to depolymerize peptide N and increase bioavailable N for both plants and microorganisms [63]. Mirroring the burning-induced increase in soil inorganic N (Fig. S9) associated with the release of NH_4_^+^ from combustion of forest biomass and surface organic matter and decreased plant uptake [64], many enriched A strategy traits were related to inorganic N uptake and transformation. These included assimilatory nitrate reduction and more widely encoded transport systems for various N species (e.g. amide, nitrate, nitrite, urea). Along with inorganic N, genes for substrate uptake were more widely encoded in the dominant soil microbiome 1 year following high-severity wildfire (Fig. S10).

**Figure 4:**
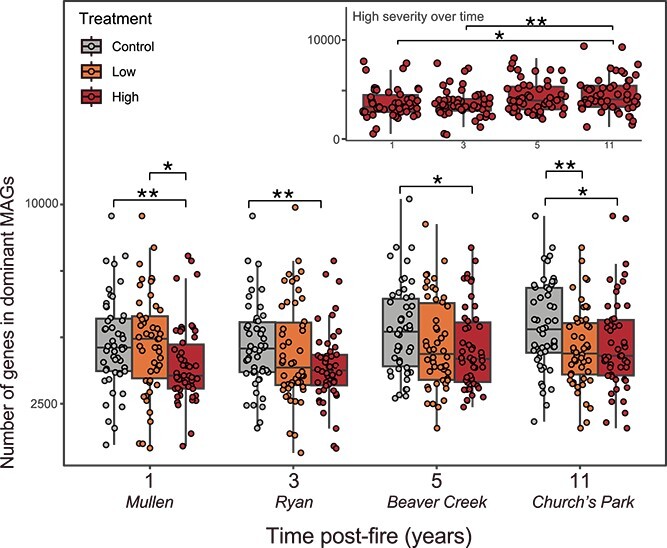
Total number of encoded genes in dominant 50 MAGs across treatments. Inset includes only high-severity treatment over time. The lower and upper hinges of the boxplots represent the 25th and 75th percentiles, respectively, and the middle line is the median. The whiskers extend from the median by 1.5× the interquartile range. Points represent individual MAGs. Significant differences between conditions indicated with asterisks as indicated by Wilcoxon rank-sum test. ^*^P < .05, ^*^^*^P < .01.

The increased genomic investment of the high-severity-fire-impacted soil microbiome into A traits suggests resource scarcity in burned soils. Evidence for resource scarcity was also supported by selection for decreased genome size across soils from all high-severity-impacted timepoints (ANOVA by treatment *P* = 5.12e−5, by time post-fire *P =* .0041, treatment × time post-fire *P* = .4804; Fig. 4, Table S6). Decreased genome size has been previously associated with both resource scarcity [65] and thermal stress [66] in soils.

### Prevalence of putative pyrophilous traits in post-wildfire soil microbiome

Severe wildfire can cause distinct short- and long-term alterations to soil, like the initial increase in soil temperature caused by heating and lasting burning-induced alterations to soil chemistry (e.g. increased pH, increase in aromaticity of soil C) [17]. Because of the unique signature of wildfire on soils, there are particular functional traits hypothesized to drive post-wildfire soil microbial succession [21, 22] that differ from those proposed in the previously discussed Y-A-S framework. These traits include the ability to persist during the initial heat pulse disturbance (“fire survival,” e.g. heat shock proteins), the ability to grow quickly and fill in newly unoccupied niche space following wildfire-induced loss of microbial biomass (Fig. S3; “fast growth”), and capacity to withstand the longer-term press disturbance of wildfire-altered soil chemistry (“post-fire environmental affinity”; e.g. ability to degrade aromatic C) [21, 22]. Although some of the functions (heat shock proteins) might align with the S strategy defined in the Y-A-S framework, the traits proposed in the traits-based fire ecology framework are more specialized for the effects of wildfire on soil microbiomes. Here, we identified the prevalence of these traits across the dominant 50 MAGs within each condition along with a subset of 23 MAGs that represented previously hypothesized putative pyrophilous taxa within the Actinobacteria *Blastococcus* (*n* = 8) and *Arthrobacter* (*n* = 10) and Proteobacteria *Massilia* (*n* = 5). This subset of 23 putative pyrophilous MAGs were all dominant in at least one of the high-severity treatments, confirming the importance of these taxa in post-wildfire soils.

**Figure 5:**
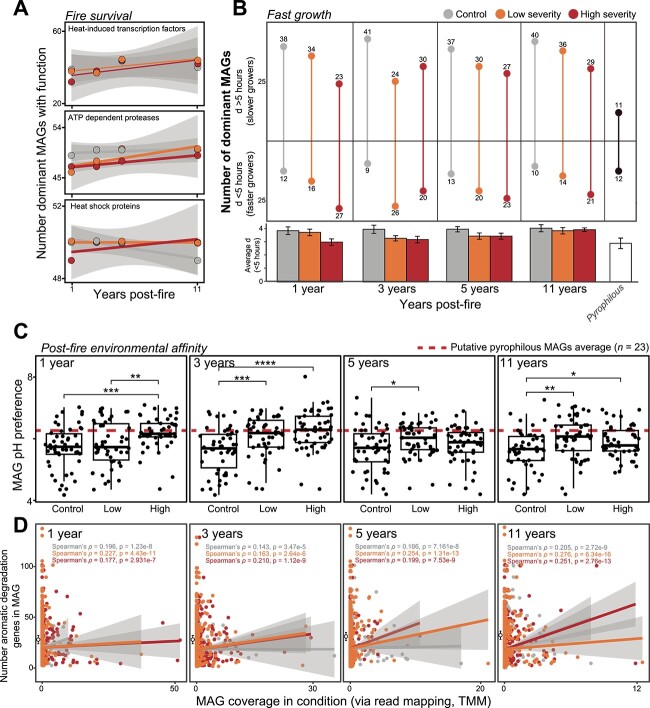
(**A**) Number of dominant MAGs for each treatment with specific “fire survival” high-temperature stress-resistant function plotted against time since fire (e.g. 1 year post-fire is samples from the Mullen fire) with linear regression lines colored by treatment. (**B**) Number of dominant MAGs (total *n* = 50) from each treatment and MAGs representing putative pyrophilous taxa (total *n* = 23) with gRodon estimated minimum doubling time (*d*) >5 or <5 h, with average *d* of MAGs <5 h indicated in below bar graphs. Error bars represent standard error of the mean. (**C**) MAG pH preference of dominant MAGs across treatments with dashed line indicating median MAG pH preference of the subset of putative pyrophilous taxa MAGs. The lower and upper hinges of the boxplots represent the 25th and 75th percentiles, respectively, and the middle line is the median. The whiskers extend from the median by 1.5× the interquartile range. Points represent individual MAGs. Significant differences between conditions indicated with asterisks as indicated by Wilcoxon rank-sum test. ^*^P < .05, ^*^^*^P < .01, ^*^^*^^*^P < .001, ^*^^*^^*^^*^P < .0001. (**D**) Individual MAG coverage in treatment (e.g. 1 year post-fire low severity) plotted against the total number of aromatic degradation genes in MAG with Spearman’s rho indicated on plot. Black-outlined, white points on each subplot show average number of aromatic degradation genes in putative pyrophilous taxa MAGs with error bars showing standard error of the mean.

There was no enrichment of fire survival traits (also known as “S strategy traits” within the Y-A-S framework) until 5 and 11 years post-fire (Fig. S6), suggesting that some S traits might be beneficial for microbes experiencing longer-term press disturbances of wildfire. Specifically, there was no significant correlation between years post-fire and the number of dominant MAGs encoding mechanisms for persisting during wildfire-induced high soil temperatures (e.g. heat shock proteins, ATP-dependent proteases for repairing denatured proteins; Fig. 5) and for tolerating desiccation stress due to short-term decreased soil moisture (Fig. S9). In the high-severity-fire-impacted soil microbiome, this initial (i.e. 1 and 3 years post-fire) lack of enriched “fire survival” traits could be due to the relatively narrow extent of the purported “Goldilocks zone”—the depth layer in the soil where temperatures increase just enough to select for microorganisms that encode heat resistance genes without killing them [8]. Here, the use of surface (0–5 cm) soils for analyses likely primarily sampled the “necromass zone” [8], or the region of the soil profile where temperatures lyse cells, causing increases in proteinaceous C and N for colonizing microorganisms to use and proliferate post-fire, and the aforementioned enrichments of A traits (e.g. aminopeptidases, Fig. 3, Fig. S8).

With the hypothesized influx of bioavailable necromass-derived C and N, the ability to grow quickly and utilize these substrates should lead to initial (i.e. 1 year post-fire) dominance of putative copiotrophs that subsequently decreases over time. Here, we used gRodon [46] to estimate individual MAG minimum doubling time via trends in CUB to assess whether these patterns persisted in the environment. We detected a clear impact of increasingly severe wildfire on the selection for taxa that could grow quickly, with more dominant MAGs in burned soils with estimated minimum doubling times <5 h (Fig. 5b). Furthermore, we observed faster average growth rates (of *d* < 5 h) in these MAGs and those representing putative pyrophilous taxa (Fig. 5b). Combined, these data highlight the importance of fast growth in the immediate post-fire soil microbiome that diminishes by ~11 years post-fire. Importantly, copiotrophs are hypothesized to grow less efficiently (i.e. lower CUE) than oligotrophs [23,67], which might lead to greater C loss from post-wildfire soils and less stable C accumulation via formation of microbial biomass.

Post-fire environmental affinity, or the ability to tolerate altered soil chemistry conditions, was evaluated by assessing the preference of dominant taxa for high pH soil environments and the genomic potential to degrade aromatic molecules, which can account for a large fraction of PyC. To quantify and compare pH preferences across the post-fire soil microbiome, we used a machine learning model using 56 predictor genes found to be predictive of bacterial pH cell preferences [47]. Overall, there was evidence of persistent (>11 years) selection for taxa that could tolerate elevated soil pH often caused by high-severity wildfire [4,68] and evident in the 3-year post-wildfire site (Fig. 5c, Fig. S9). Both fire treatment (via ANOVA, *P* = 2.31e−9) and time post-fire (*P* = .0269) had a significant effect on the dominant MAG pH preference, with a significant interactive effect (*P* = .0135) indicating that the effect of fire treatment differed depending on the time post-fire (Table S7). This trait was also present across the putative pyrophilous MAGs, with a median pH preference similar to that calculated for dominant MAGs from 1 and 3 years after high-severity wildfire.

In contrast to the selection of bacteria with higher pH preferences, there was no clear impact of wildfire on the functional potential of the soil microbiome to degrade aromatic C, both in the dominant MAGs in burned soils and the MAGs representing putative pyrophilous taxa, as even in control soils more dominant MAGs encoded more aromatic C degradation genes (Fig. 5d). However, within 3 years of high-severity wildfire, MAGs encoding more genes for degrading aromatics were more strongly enriched in burned soils as compared to control (higher Spearman rho values; Fig. 5d). This supports previous work [69] hypothesizing that more labile PyC fractions (i.e. oxygenated or low molecular weight components [70]) are initially mineralized and depleted, followed by use of more aromatic forms of PyC. PyC is traditionally considered to be relatively recalcitrant to microbial degradation and more work is needed to assess implications of microbial degradation of PyC on post-wildfire ecosystem C budgets [71].

Together, this dataset confirms the importance of fast growth and affinity for high pH soils in the post-wildfire soil microbiome. Given that the putative pyrophilous taxa studied here (e.g. *Blastococcus*, *Arthrobacter*, *Massilia*) have been documented across diverse fire-adapted ecosystems, including CO coniferous forests [6, 7], CA chaparral [25], boreal forests [24], and Mediterranean holm oak forests [72,73], we propose that these traits might be globally relevant in the post-wildfire soil microbiome and might help inform and predict wildfire impact on microbially mediated biogeochemical cycling.

### Implications of post-wildfire trait selection for ecosystem C cycling

Accurately predicting post-wildfire ecosystem biogeochemistry is challenging due to heterogeneity across ecosystems, difficulties upscaling field-generated data, and key knowledge gaps introducing uncertainty (e.g. wildfire impacts to feedbacks between above- and belowground processes relevant to C fluxes) [74]. This is especially pertinent as climate change and human intervention is causing an increase in the severity, frequency, and extent of wildfires across the globe [75–77], resulting in hampered ecosystem resilience [78,79] that can lead to slow vegetation recovery or ecosystem conversions [80]. Classifying key pyrophilous soil microbial taxa and identifying traits that define and characterize the post-wildfire soil microbiome across ecosystems could help distill complex microbial data and increase predictability of post-wildfire soil microbial dynamics and their biogeochemical consequences. Here, we show that wildfire initially selects for taxa that are copiotrophic and can grow quickly to occupy empty niche space (Fig. 5b) and, up to a decade post-fire, taxa that invest in resource acquisition strategies (Fig. 3). These findings suggest increased post-wildfire soil C losses induced by these shifting microbial traits as both copiotrophs and microorganisms that invest in resource acquisition traits are hypothesized to grow less efficiently (i.e. lower CUE) [23,62,67]. Understanding the linkages between wildfire, soil microbial life history strategies, and CUE is imperative as microbial growth efficiency is positively correlated with soil C stocks [81] and wildfire-induced shifts in community CUE could influence the post-wildfire ecosystem C budget. Combined, this work suggests that severe wildfire selects for a soil microbiome that largely encodes traits that could lead to increased soil C losses for a decade following wildfire.

## Conclusion

Enhancing our understanding of linkages between life history strategy traits and post-disturbance soil microbial succession can enhance the predictability of how disturbances alter microbially mediated ecosystem biogeochemistry. Here, we used a genome-resolved metagenomics approach to generate a MAG catalog (FiRE-db) representing a post-wildfire coniferous forest soil microbiome over time (1–11 years) and disturbance severity (control, low- and high-severity wildfire). This comprehensive set of MAGs from wildfire-impacted soils provided insights into whether pyrophilous taxa previously identified using amplicon sequencing have specific life history strategy traits that enable their persistence during or following a wildfire disturbance. Severe wildfire resulted in a short-term enrichment in pyrophilous taxa that encoded the genomic potential for fast growth and preference for elevated pH soils. In the longer term, taxa that invested in resource acquisition were enriched in severely burned soils. Both fast microbial growth and investment in acquiring diverse resources from the surrounding environment could result in enhanced soil C losses following wildfire, but increased investment in diverse peptidases may help produce bioavailable N for plant reestablishment and thus be a critical component of the N cycle following wildfire. Therefore, the application of trait-based, life history strategy frameworks to disentangle post-disturbance microbial ecology could provide invaluable insight and predictability into complex soil microbial succession dynamics and changes in terrestrial biogeochemistry.

## Supplementary Material

Nelsonetal_Supplementary_Information_ycae108

## Data Availability

The metagenomic reads and bacterial MAGs reported in this paper have been deposited in National Center for Biotechnology Information (NCBI) BioProject PRJNA682830. NCBI Accession numbers for metagenomic reads and MAGs are included in the Supplemental Data.
