# Correction to: Wildfire impact on soil microbiome life history traits and roles in ecosystem carbon cycling

**DOI:** 10.1093/ismeco/ycag202

**Published:** 2026-07-27

This is a correction to: Amelia R Nelson, Charles C Rhoades, Timothy S Fegel, Holly K Roth, Marcos V Caiafa, Sydney I Glassman, Thomas Borch, Michael J Wilkins, Wildfire impact on soil microbiome life history traits and roles in ecosystem carbon cycling, *ISME Communications*, Volume 4, Issue 1, January 2024, ycae108, https://doi.org/10.1093/ismeco/ycae108

In the originally published version of this manuscript, the supplementary data section was incomplete.

The omitted supplementary material has been added to this correction notice.

This error has been corrected only in this correction notice to preserve the version of record.

