# Supplementary material for: Wildfire impact on soil microbiome life history traits and roles in ecosystem carbon cycling
Source: ISME Commun. 2024 Aug 27;4(1):ycae108. doi: 10.1093/ismeco/ycae108 (PMC11831523; doi:10.1093/ismeco/ycae108)
Supplement: Nelsonetal_Supplementary_Information_ycae108 [file nelsonetal_supplementary_information_ycae108.pdf]

Supplementary Information for
Wildfire impact on soil microbiome life history traits and roles in ecosystem carbon cycling

Amelia R. Nelson[1], Charles C. Rhoades[2], Timothy S. Fegel[2], Holly K. Roth[1], Marcos V. Caiafa[3], Sydney I. Glassman[3], Thomas Borch[1,4], Michael J. Wilkins[1]*

[1]Department of Soil and Crop Sciences, Colorado State University, Fort Collins, CO (USA) 80523
[2]Rocky Mountain Research Station, United States Forest Service, Fort Collins, CO (USA) 80526
[3]Department of Microbiology and Plant Pathology, University of California Riverside, Riverside, CA (USA) 92521
[4]Department of Chemistry, Colorado State University, Fort Collins, CO (USA) 80523

Corresponding Author (*):
Dr. Michael J. Wilkins
Colorado State University
Department of Soil and Crop Sciences
1170 Campus Delivery
Fort Collins, CO 80523-1170
Mike.wilkins@colostate.edu

This PDF file includes:

Supplementary Text	2-3
Supplementary Figures 1-13	4-17
Supplementary Table 1-6	18-20
Supplementary Dataset Information	21
References	22

Other supporting materials for this manuscript include the following:

Supplementary Data

Supplementary text

Broad overview of life history trait profiles in FiRE-db

Of the profiled traits by *microTrait* (*n* = 189), 29 were encoded by the majority of MAGs (80% or >661 MAGs). These traits largely fell under the A strategy (18 of the 29), including functions for complex carbohydrate depolymerization (e.g., xylan, chitin, and cellulose). The trait most widely encoded in the MAG catalog (824 of the 825 MAGs) was for protein degradation, likely reflecting abundant microbial necromass in soil C (estimated at ~30-40% of SOM(1–3)) and the likely ubiquitous degradation of microbial necromass by active soil microbes. The remaining 11 widely encoded functions were all associated with S strategies, including genes for enduring temperature fluctuations (both high and low temperatures), desiccation, and pH stress. S strategy traits were also more widely distributed within the MAG catalog (**Fig. S13**; average of 489 MAGs encoding each trait vs. 304 MAGs for A traits), indicating the evolutionary importance of environmental responsiveness to fluctuating conditions within soils. Combined, these data shows that functional potential with the soil microbial communities broadly aligns with A and S life history strategies. Moreover, this results aligns with a large-scale biogeography analysis revealing that the soil microbiome of coniferous forests generally fall within these life history strategies with variation caused by soil pH(4). The forests studied here were also greatly impacted by severe mountain pine beetle outbreak in ~2005, with control sites having ~75% average overstory tree mortality(5,6), potentially contributing to overall lower resource availability in these soils(7) and resulting in increased genomic investment into acquiring diverse resources (A strategy).

Visualizing differences in MAG trait allocations in multivariate space, we observed separation largely based on taxonomy, with much of the dissimilarity explained by bacterial phyla (PERMANOVA $R^2 = 0.45$, $p = 0.001$) and class (PERMANOVA $R^2 = 0.54$, $p = 0.001$) (**Fig. S14**). Here, despite recent critiques(8), bacterial taxonomy to the class level is a large driver of genomic trait allocation. MAGs affiliated with the Proteobacteria and Actinobacteria had among the highest number of encoded traits (normalized by number of phyla MAGs in dataset; **Fig. S15**) for both A and S strategies, likely lending to their general high abundance in soils(9). In contrast, MAGs affiliated with the Acidobacteria preferentially encoded S traits over A traits, while Verrucomicrobia MAGs displayed the opposite trend. Overall, these analyses point to fine-scale interphylum differences in genomic allocation to various life history strategies.

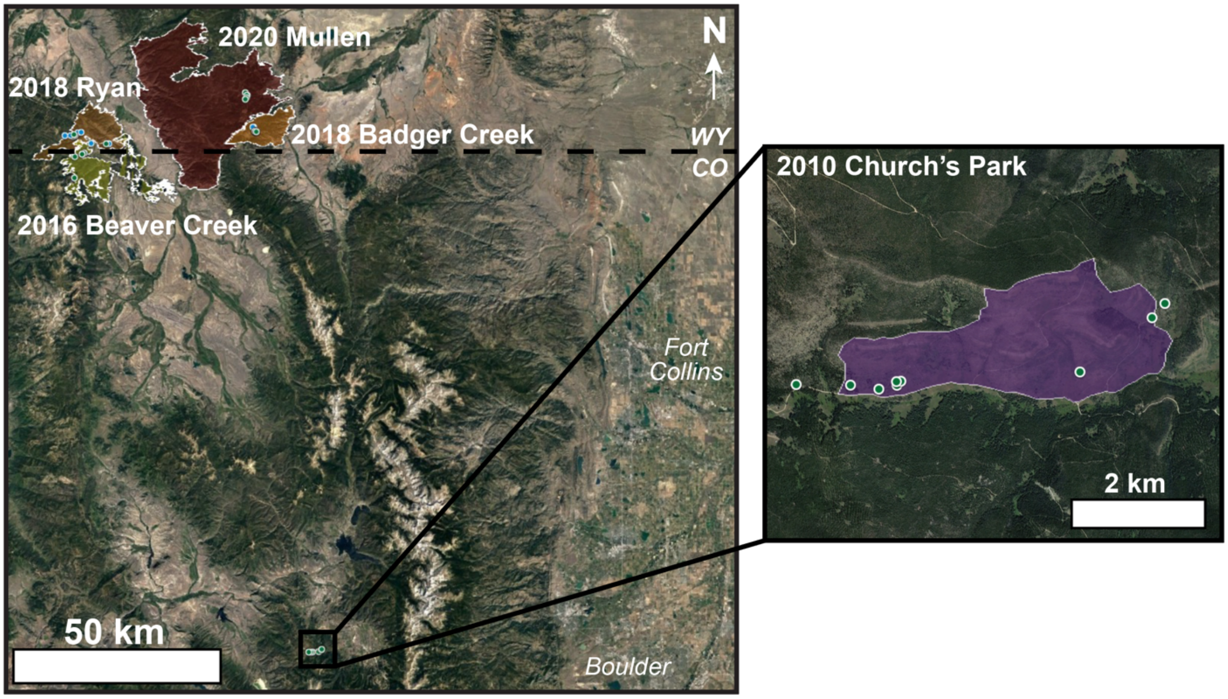

Fig. S1. Sampled burn scars. At the time of sampling (2021), the chronosequence of burn scars spanned from 1 to 11 years post-fire. Points represent different sampling locations.

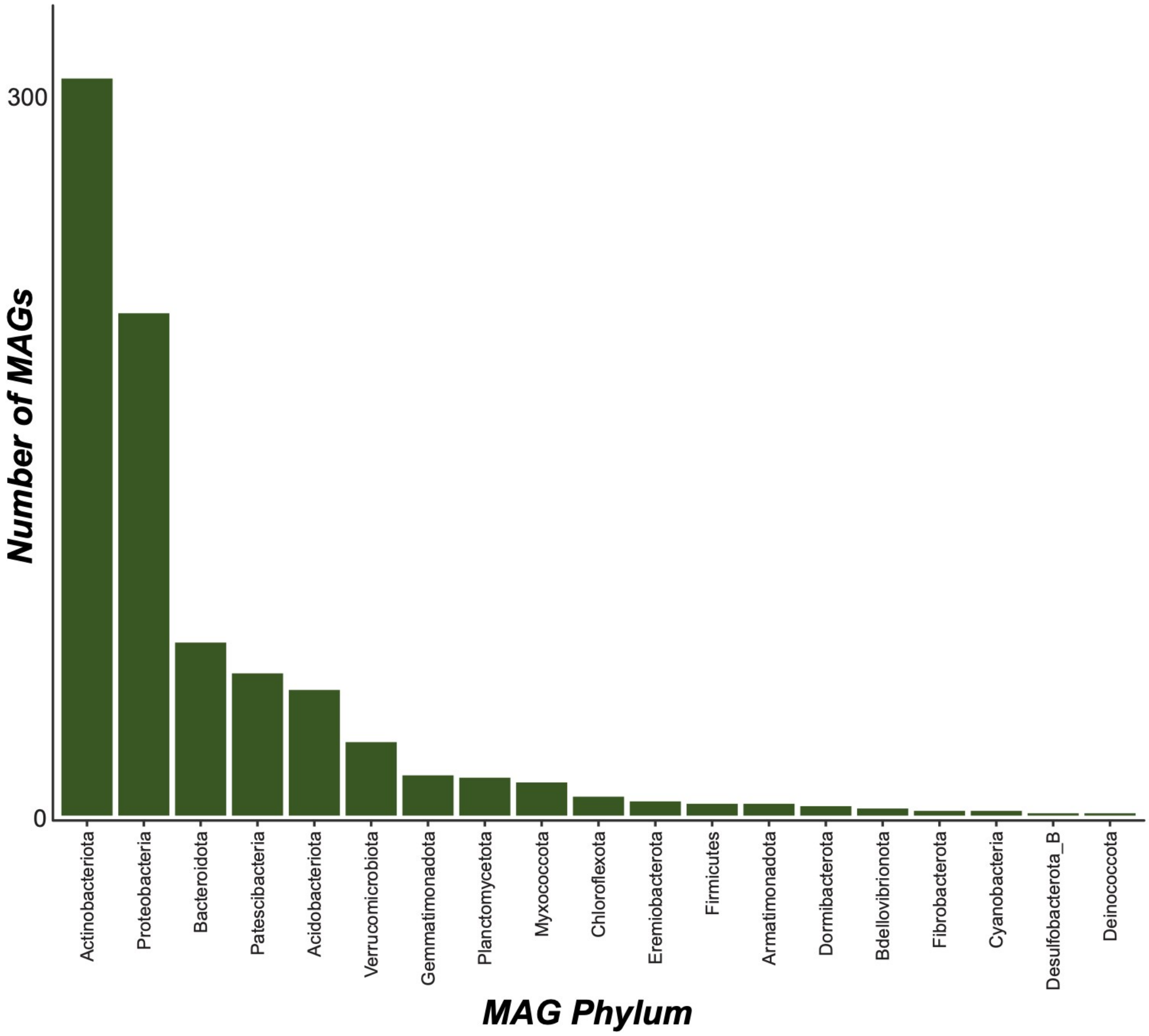

Fig. S2. Bacterial phyla overview of metagenome-assembled genomes (MAGs) (n = 825).

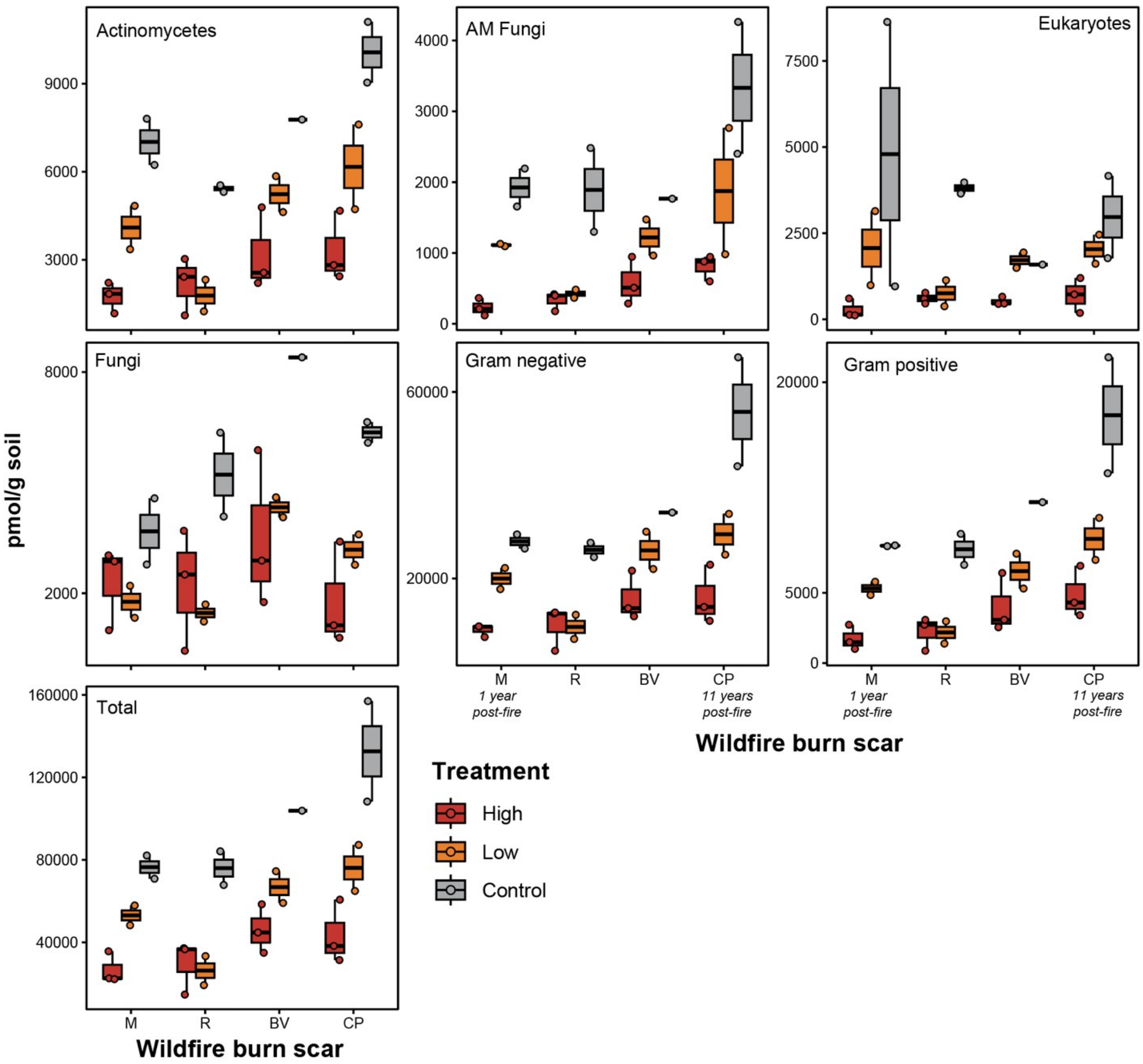

Fig. S3. Microbial biomass (from PLFA analyses) from across treatments and time post wildfire. M = Mullen fire (sampled 1 year post-fire), R = Ryan fire (3 years), BV = Beaver Creek fire (5 years), and CP = Church's Park fire (11 years). The lower and upper hinges of the boxplots represent the 25th and 75th percentiles, respectively, and the middle line is the median. The whiskers extend from the median by 1.5x the interquartile range.

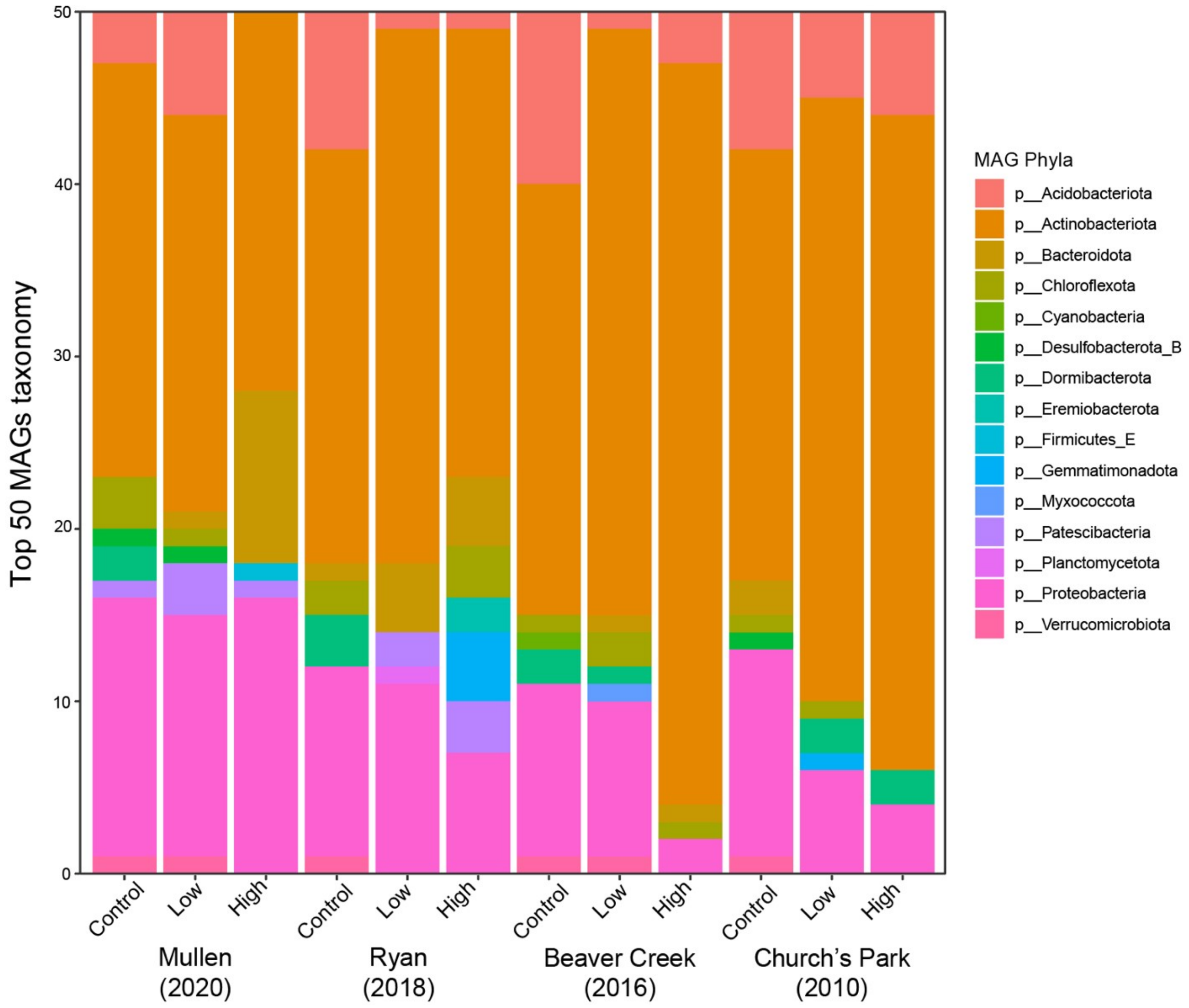

Fig. S4. Phyla distribution of MAGs that were the top 50 most abundant in each condition (determined via read mapping to MAG catalog).

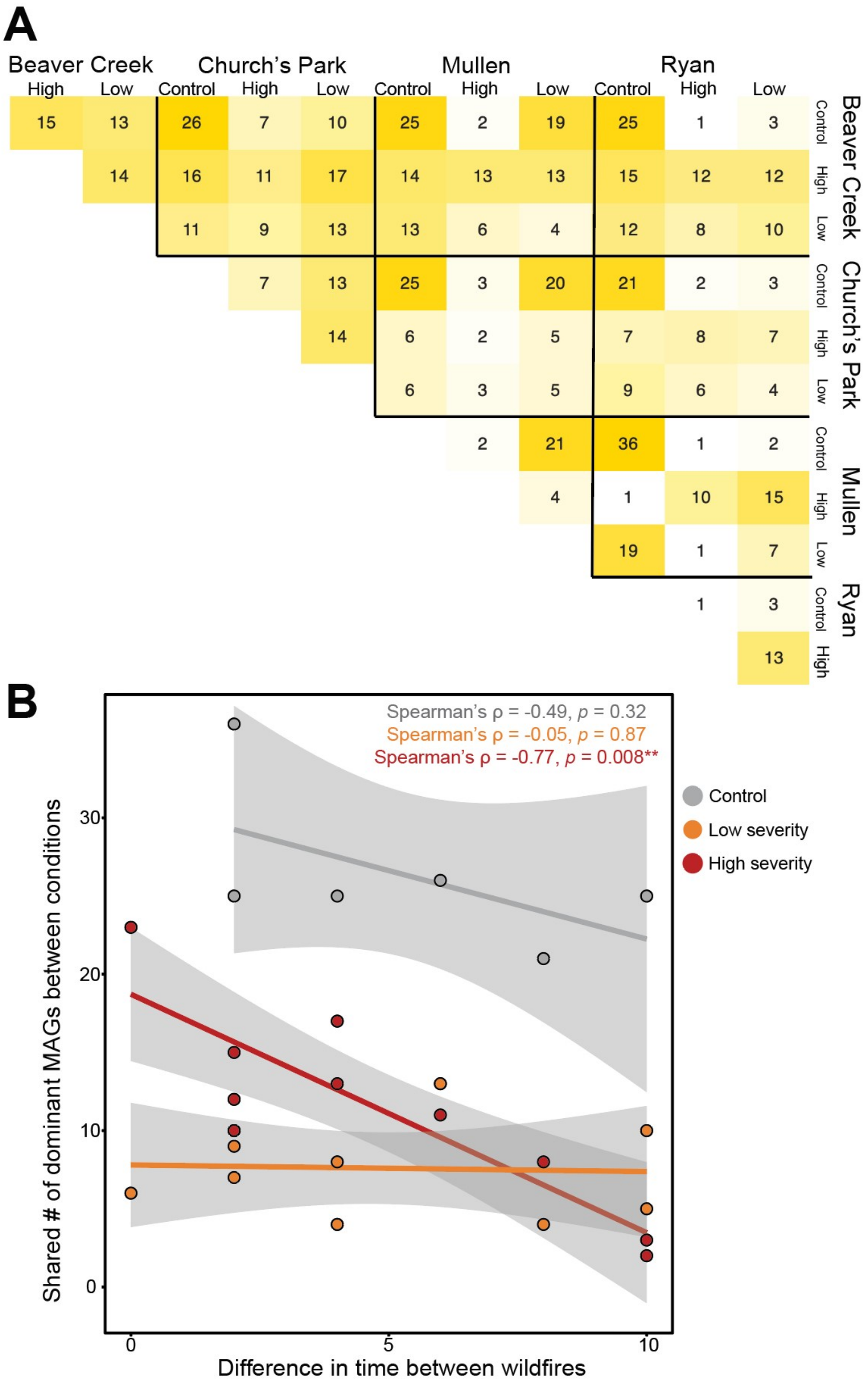

Fig. S5. (A) Number of dominant MAGs shared between each condition. (B) Number of shared dominant MAGs between conditions plotted against the difference in time between treatment of wildfires. For example, the high severity points plotted at 10 years are showing the number of shared dominant MAGs between the Mullen (2020) and Church's Park fire (2010). Correlation and significance were assessed using the two-sided Spearman rho test.

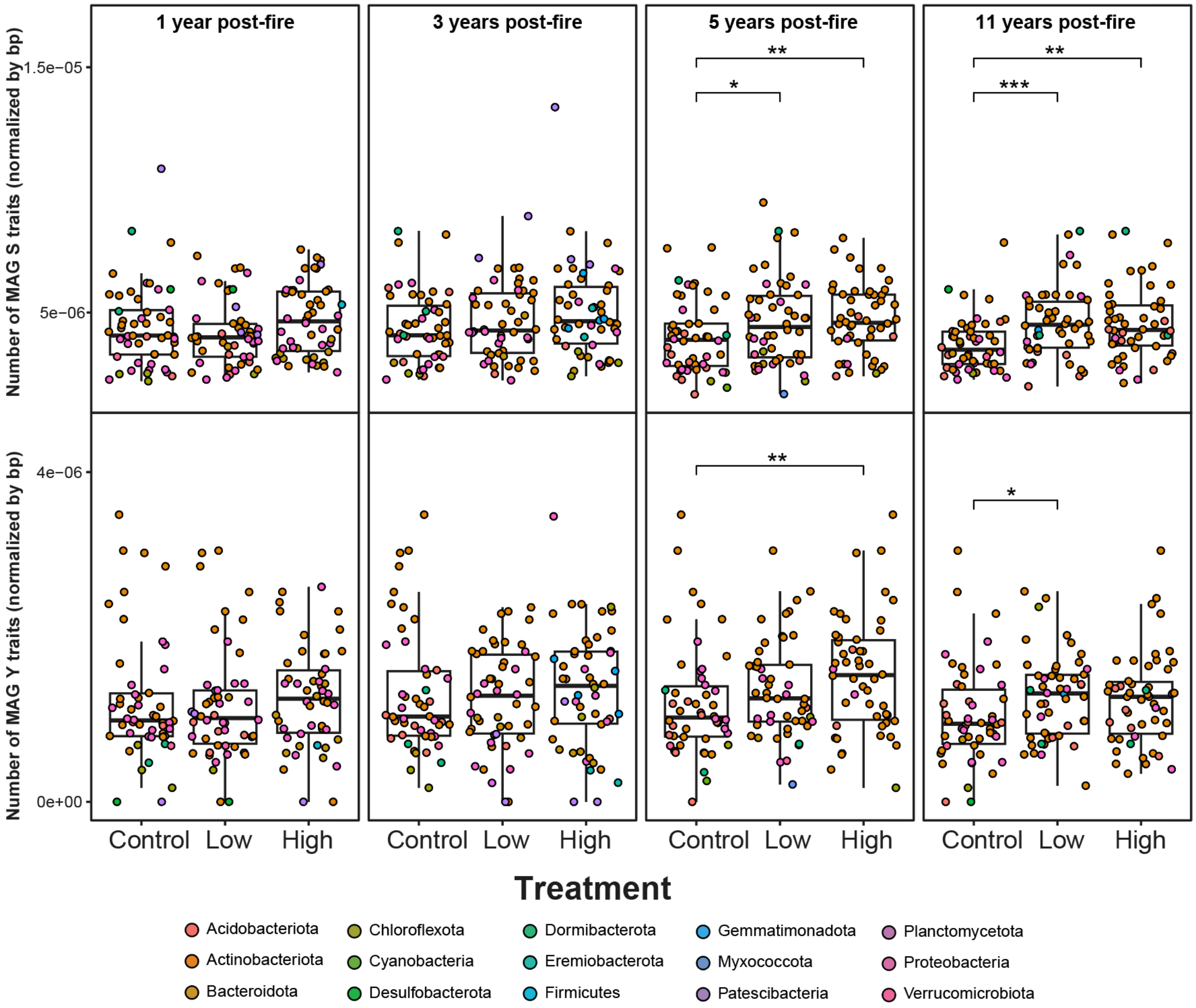

Fig. S6. Genome sized normalized investment into *microTrait* assigned resource use (Y; bottom) and stress tolerance (S; top) traits of top 50 most dominant MAGs across conditions. The lower and upper hinges of the boxplots represent the 25th and 75th percentiles, respectively, and the middle line is the median. The whiskers extend from the median by 1.5x the interquartile range. Points represent individual MAGs. Significant differences between conditions indicated with asterisks as indicated by Wilcoxon rank-sum test. *p<0.05, **p<0.01, ***p<0.001.

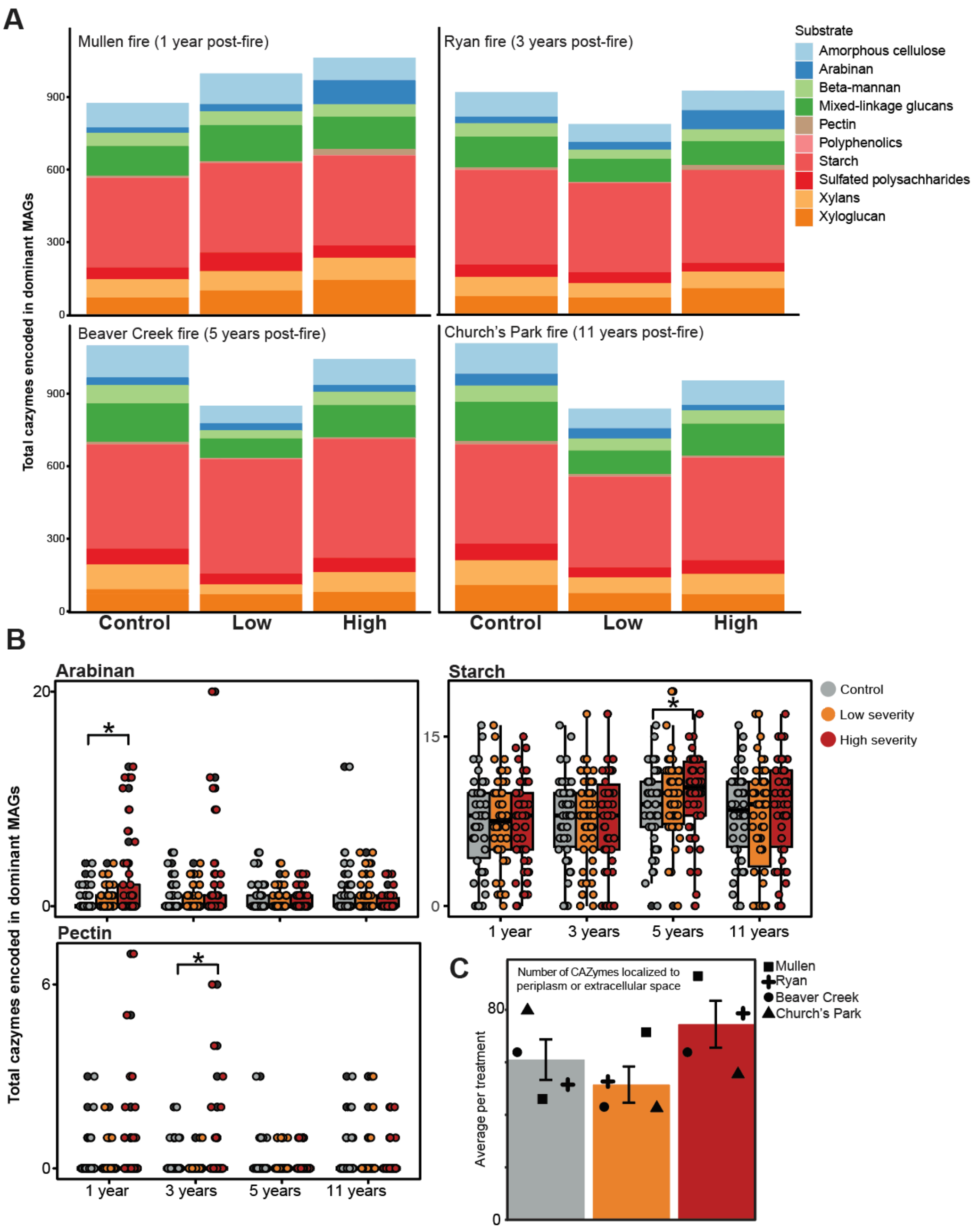

Fig. S7. (A) Total number of CAZymes encoded in all 50 dominant MAGs across conditions, colored by substrate that CAZyme acts upon. **(B)** Boxplot of CAZymes for targeting specific substrates encoded in dominant MAGs across treatments. The lower and upper hinges of the boxplots represent the 25th and 75th percentiles, respectively, and the middle line is the median. The whiskers extend from the median by 1.5x the interquartile range. Points represent individual MAGs. Significant differences between conditions indicated with asterisks as indicated by Wilcoxon rank-sum test. **p*<0.05, ***p*<0.01. **(C)** Average number of CAZymes localized to

periplasm or extracellular space, with individual points representing number from each wildfire site.

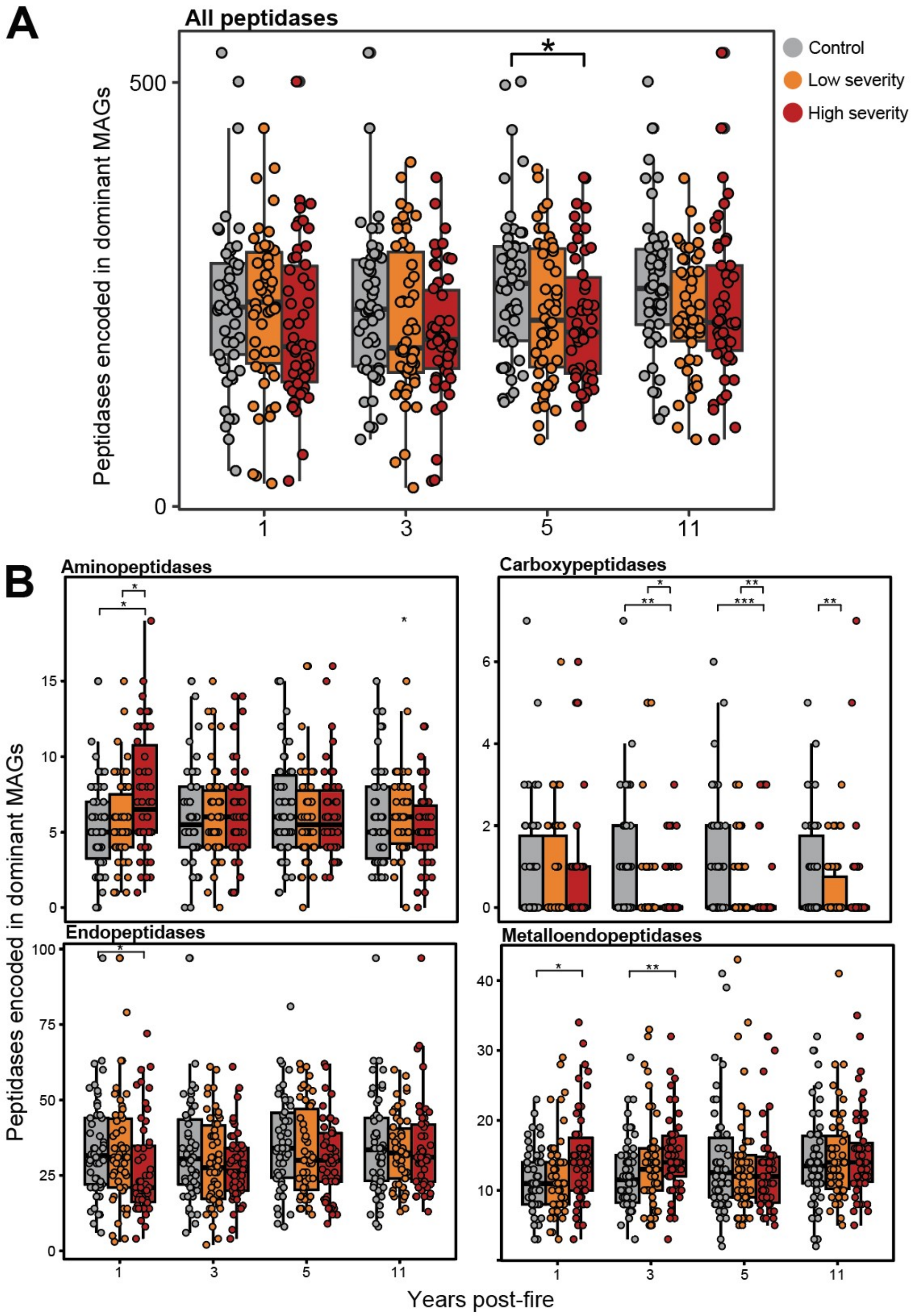

Fig. S8. (A) Total number of peptidases encoded in all 50 dominant MAGs across conditions. **(B)** Boxplot specific types of peptidases across treatments. The lower and upper hinges of the boxplots represent the 25th and 75th percentiles, respectively, and the middle line is the median. The whiskers extend from the median by 1.5x the

interquartile range. Points represent individual MAGs. Significant differences between conditions indicated with asterisks as indicated by Wilcoxon rank-sum test. *$p<0.05$, **$p<0.01$, ***$p<0.001$.

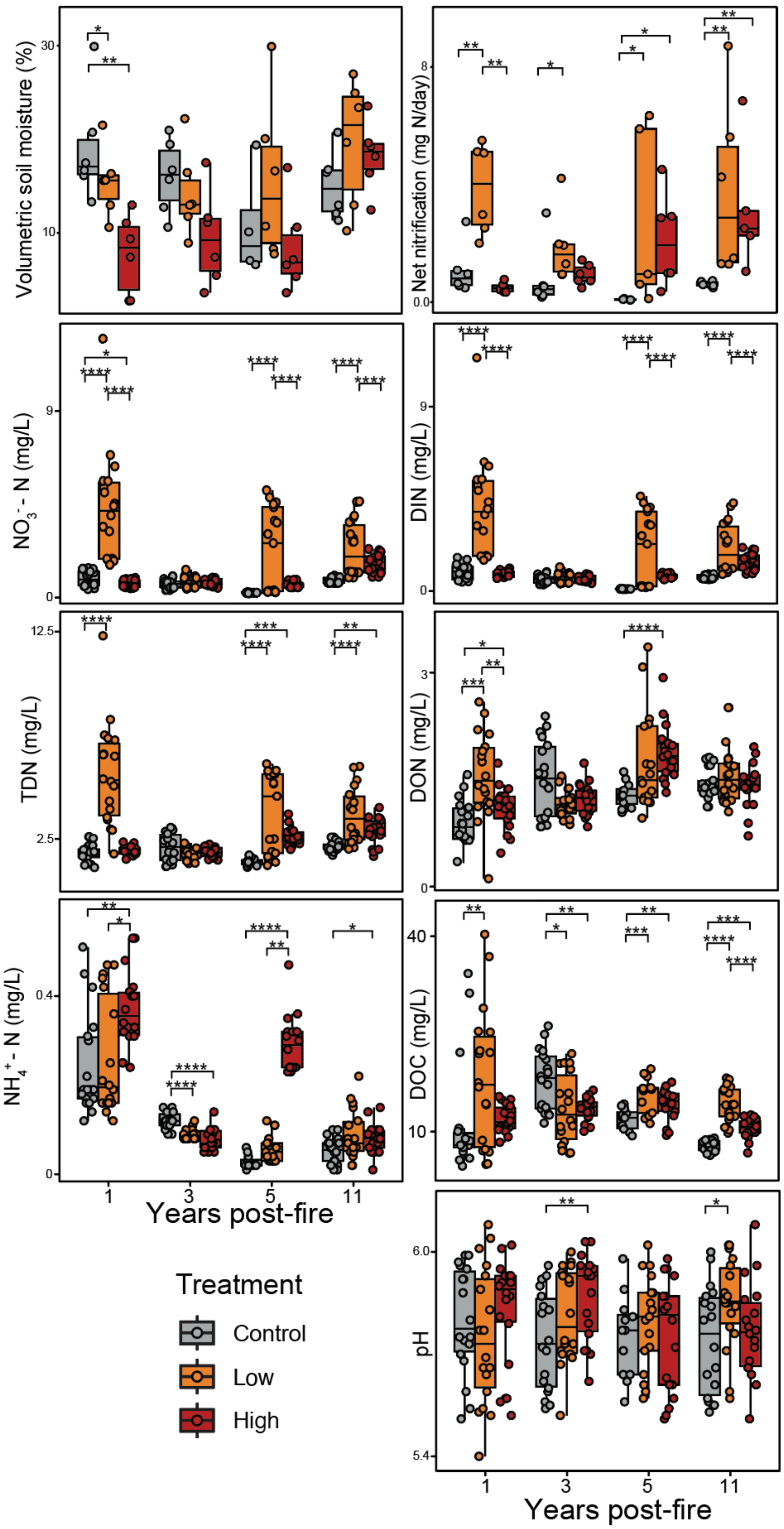

Fig. S9. Bulk soil data from water extracts and laboratory incubations. The lower and upper hinges of the boxplots represent the 25th and 75th percentiles, respectively, and the middle line is the median. The whiskers extend from the median by 1.5x the interquartile range. Jittered points represent individual samples. Significant differences between conditions indicated with asterisks as indicated by Wilcoxon rank-sum test. *p<0.05, **p<0.01, ***p<0.001, ****p<0.0001.

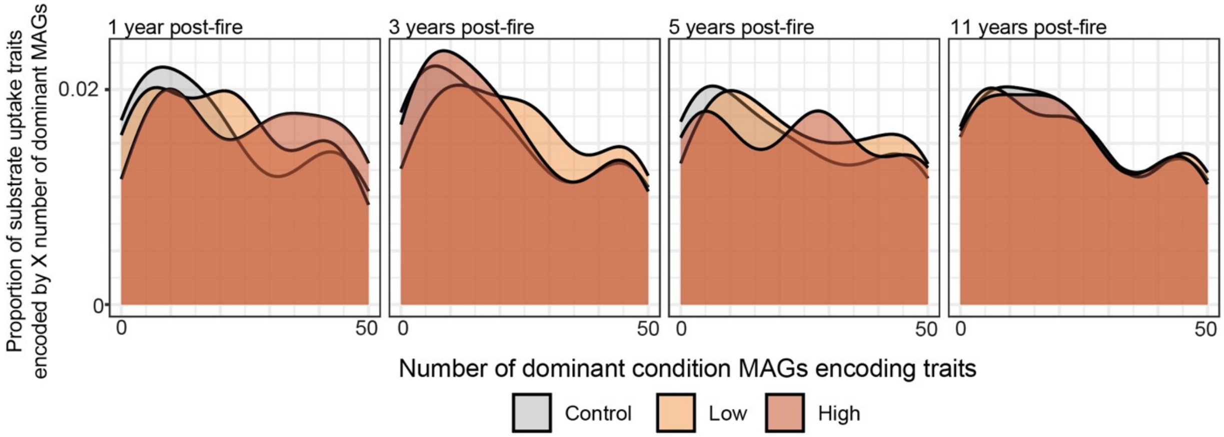

Fig. S10. Density plot showing the number of resource acquisition ‘substrate uptake’ traits that have a given number of treatment dominant MAGs that encode them, showing the enrichment of these traits in the dominant soil microbiome 1 year after high severity wildfire.

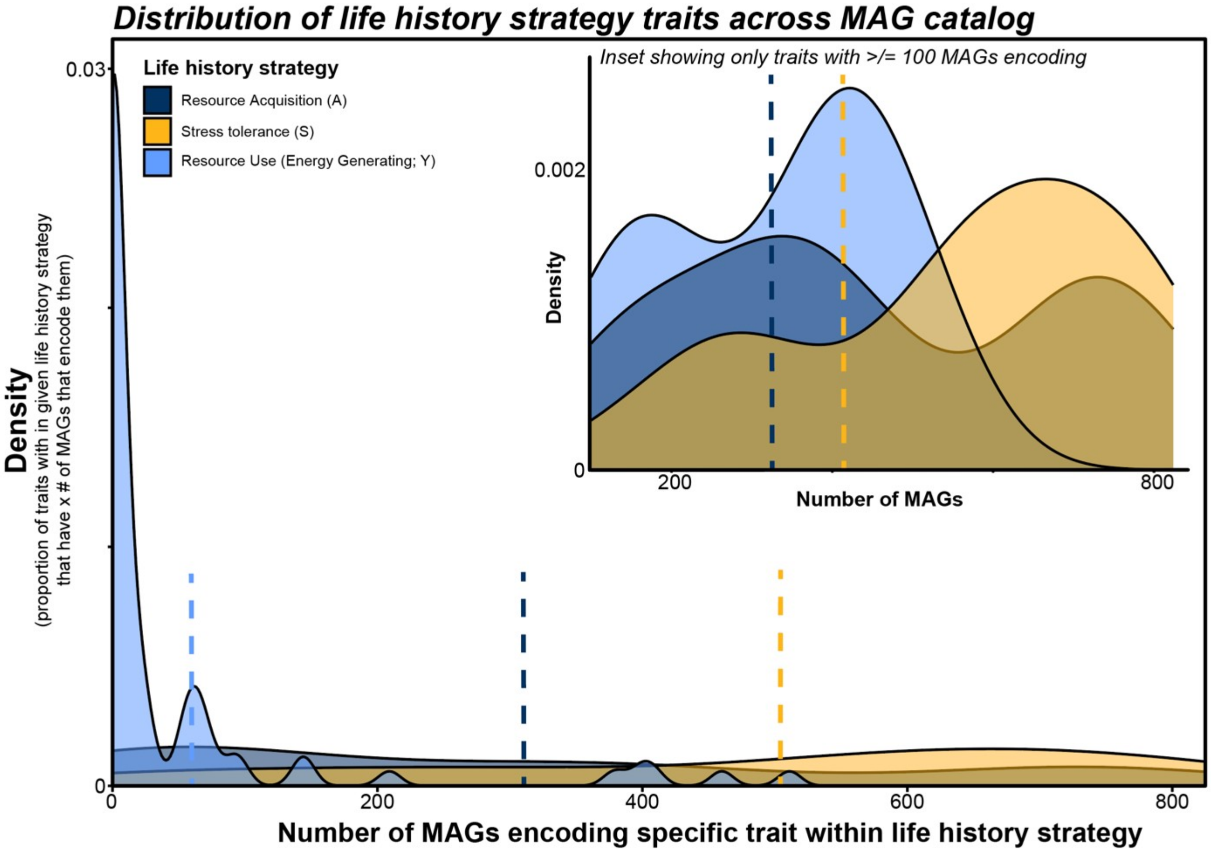

Fig. S11. Density plot showing the number of traits within a given life history strategy (Y, A, S) that have a given number of MAGs that encode them. Dashed lines show average number of MAGs that encode each trait within the given life history strategy.

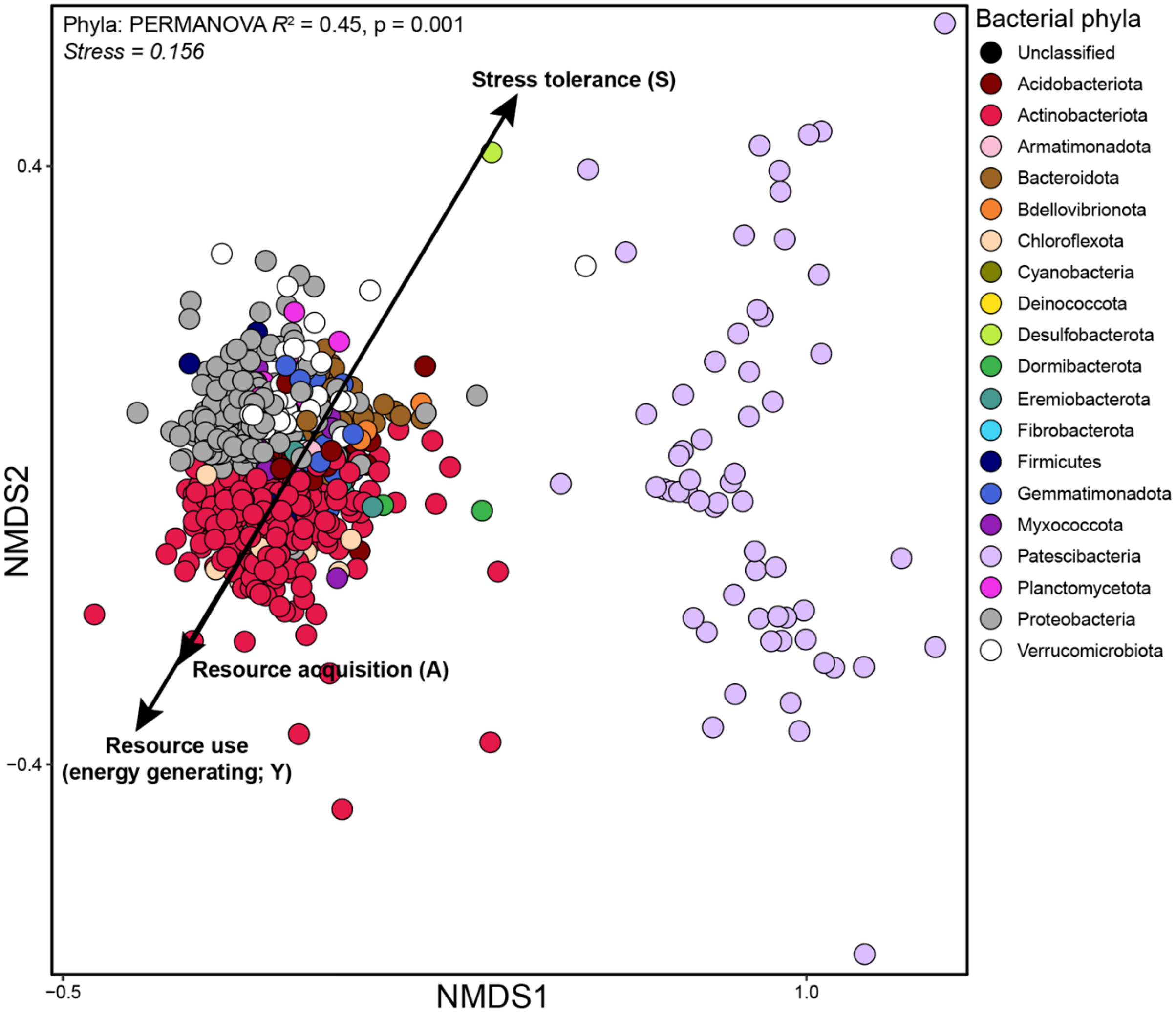

Fig. S12. Non-metric multidimensional scaling (NMDS) ordination of Bray-Curtis dissimilarity between MAGs based on MAG Y-A-S profiles generated from *microTrait*. Arrows indicate life history strategies largely driving differences.

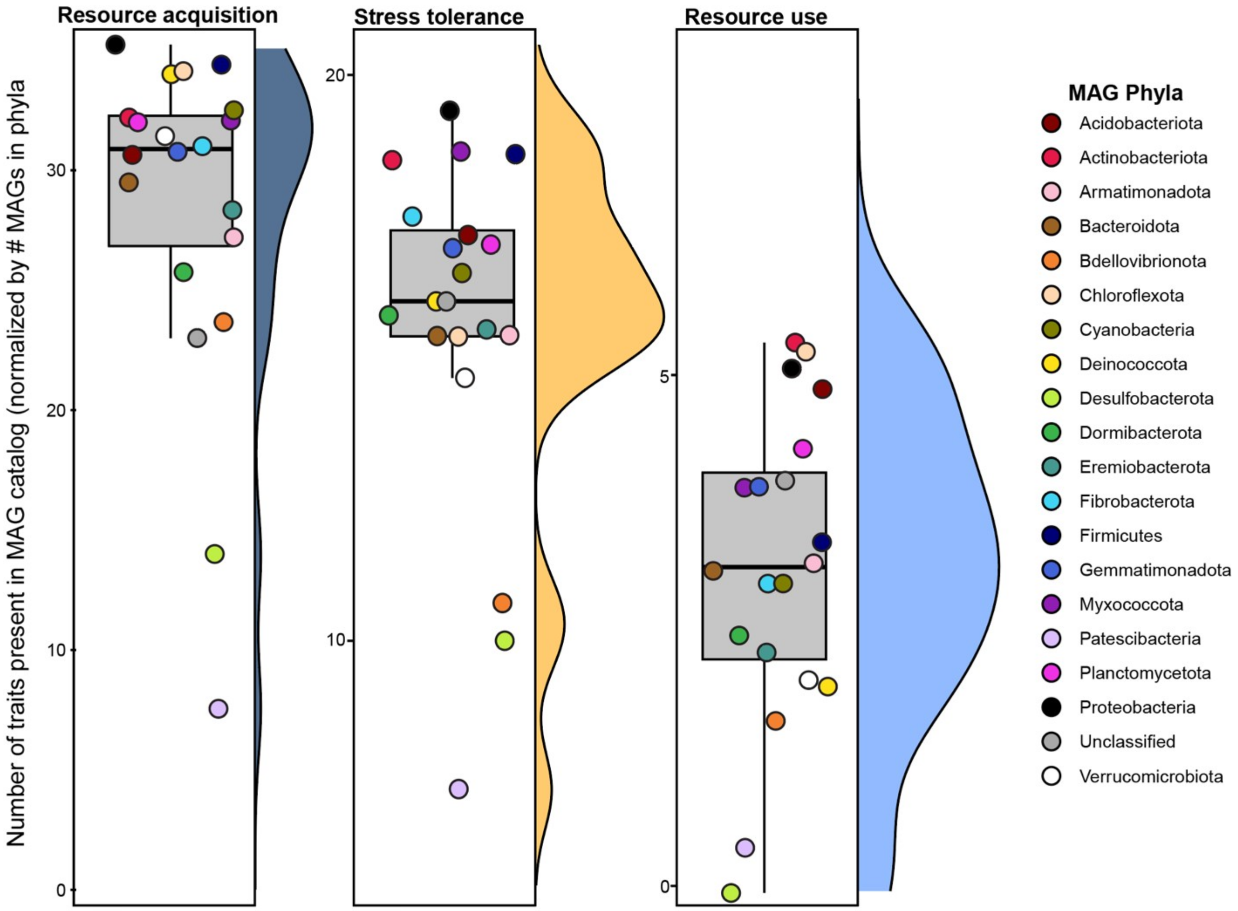

Fig. S13. Boxplots showing number of traits (via *microTrait*) encoded by MAGs classified as each bacterial phyla in each life history strategy, normalized by number of MAGs within each phylum for comparison across all phyla. The lower and upper hinges of the boxplots represent the 25th and 75th percentiles, respectively, and the middle line is the median. The whiskers extend from the median by 1.5× the interquartile range. Density plots that represent data to the right of each boxplot.

Treatment	1 year	3 years	5 years	11 years
**Control**	*3*	*3*	*3*	*3*
**Low**	*9*	*3*	*3*	*3*
**High**	*9*	*3*	*3*	*3*

Table S1. Details of samples used for metagenomic sequencing. Note that the 1 year post-fire time point has 6 more metagenomes due to the use of metagenomes from Nelson et al. (2022)(10).

Fire	Treatment	Min coverage	Max coverage
**Mullen**	**Control**	0.207	9.825
	**Low**	0.383	37.363
	**High**	2.995	52.010
**Ryan**	**Control**	0.274	35.529
	**Low**	1.205	28.147
	**High**	2.916	29.463
**Beaver Creek**	**Control**	0.274	17.008
	**Low**	0.445	21.152
	**High**	0.122	10.670
**Church's Park**	**Control**	0.312	7.822
	**Low**	0.445	21.152
	**High**	0.563	11.864

Table S2. Coverage ranges of top 50 most dominant MAGs across conditions.

**PERMANOVA**	Number of Permutations: 999				
	DF	Sum of Sqs	R^2	F	p
Treatment	2	1.7338	0.11486	2.3780	0.001***
Time post-fire	3	1.6334	0.10821	1.4935	0.005**
Treatment*time post-fire	6	2.9779	0.19728	1.3614	0.004**
Residual	24	8.7495	0.57964		

Table S3. PERMANOVA (using adonis2) on Bray-Curtis dissimilarity testing effect of sampled burn treatment (control, low, high burn severity) and time post-fire on microbial community composition (via MAG relative abundances).

PERMANOVA	Number of Permutations: 999			
Fire (time post-fire)	Sum of Sqs	R^2	F	p
Mullen (1 yr)	1.4140	0.42371	2.2057	0.014*
Ryan (3 yr)	1.4034	0.41152	2.0979	0.019*
Beaver Creek (5 yr)	0.9076	0.26084	1.0587	0.356
Church’s Park (11 yr)	0.9867	0.30508	1.317	0.111

Table S4. PERMANOVA (using adonis2) on Bray-Curtis dissimilarity testing effect of sampled burn treatment (control, low, high burn severity) on microbial community composition (via MAG relative abundances), separated by fire site.

**ANOVA**	**Effect of treatment and time post-fire on dominant MAG encoded A traits**				
	**Sum of Sqs**	**Mean sq**	**F**	**p**	**η²**
Treatment	2.82e-10	1.41e-10	23.060	2.23e-10***	0.09
Time post-fire	5.4e-11	1.795e-11	2.933	0.03292*	0.06
Treatment*time post-fire	1.18e-10	1.965e011	3.211	0.00413**	0.02
	**Effect of treatment and time post-fire on dominant MAG encoded Y traits**				
Treatment	2.55e-12	1.27e-12	2.961	0.0526	0.02
Time post-fire	3.010e-12	1.004e-12	2.337	0.0727	0.02
Treatment*time post-fire	2.38e-12	3.968e-13	0.923	0.4776	8.91e-3
	**Effect of treatment and time post-fire on dominant MAG encoded S traits**				
Treatment	3.84e-11	1.92e-11	8.845	0.000164***	0.02
Time post-fire	1.16e-11	3.876e-12	1.786	0.14866	0.00
Treatment*time post-fire	1.97e-11	3.276e-12	1.509	0.17266	0.00

Table S5. Factorial ANOVA testing the effect of sampled burn treatment (control, low, high burn severity) and time post-fire on the normalized number of encoded A, Y, and S traits in dominant MAGs.

ANOVA	Effect of treatment and time post-fire on dominant MAG # of encoded genes			
	Sum of Sqs	Mean sq	F	p
Treatment	5.5e7	2.7e7	10.047	5.12e-5***
Time post-fire	3.7e7	1.2e7	4.468	0.0041**
Treatment*time post-fire	1.52e7	2.5e6	0.919	0.4804

Table S6. Factorial ANOVA testing the effect of sampled burn treatment (control, low, high burn severity) and time post-fire on the number of genes in dominant MAGs.

ANOVA	Effect of treatment and time post-fire on dominant MAG pH preference			
	Sum of Sqs	Mean sq	F	p
Treatment	16.59	8.294	20.572	2.31e-9***
Time post-fire	3.73	1.244	3.085	0.0269*
Treatment*time post-fire	6.53	1.089	2.701	0.0135*

Table S7. Factorial ANOVA testing the effect of sampled burn treatment (control, low, high burn severity) and time post-fire on the estimated pH preference of dominant MAGs.

Supplementary Data Sheet A. All sample metadata and associated chemistry data.

Supplementary Data Sheet B. Details about metagenomic assemblies.

Supplementary Data Sheet C. Information about all medium- and high-quality MAGs included in FiRE-db.

Supplementary Data Sheet D. FiRE-db MAG relative abundance across samples calculated from read mapping.

Supplementary Data Sheet E. Trait profiles (presence/absence) across FiRE-db.

Supplementary Data Sheet F. List of representative dominant MAGs in all conditions and putative pyrophilous taxa MAGs.

References

1. Liang C, Amelung W, Lehmann J, Kästner M. Quantitative assessment of microbial necromass contribution to soil organic matter. Global Change Biology. 2019;25(11):3578–90.

2. Wang B, An S, Liang C, Liu Y, Kuzyakov Y. Microbial necromass as the source of soil organic carbon in global ecosystems. Soil Biology and Biochemistry. 2021 Nov 1;162:108422.

3. Angst G, Angst Š, Frouz J, Jabinski S, Jílková V, Kukla J, et al. Stabilized microbial necromass in soil is more strongly coupled with microbial diversity than the bioavailability of plant inputs. Soil Biology and Biochemistry. 2024 Mar 1;190:109323.

4. Piton G, Allison SD, Bahram M, Hildebrand F, Martiny JBH, Treseder KK, et al. Life history strategies of soil bacterial communities across global terrestrial biomes. Nat Microbiol. 2023 Nov;8(11):2093–102.

5. Kayes LJ, Tinker DB. Forest structure and regeneration following a mountain pine beetle epidemic in southeastern Wyoming. Forest Ecology and Management. 2012 Jan 1;263:57–66.

6. Rhoades CC, Hubbard RM, Hood PR, Starr BJ, Tinker DB, Elder K. Snagfall the first decade after severe bark beetle infestation of high-elevation forests in Colorado, USA. Ecological Applications. 2020;30(3):e02059.

7. Trahan NA, Dynes EL, Pugh E, Moore DJP, Monson RK. Changes in soil biogeochemistry following disturbance by girdling and mountain pine beetles in subalpine forests. Oecologia. 2015 Apr 1;177(4):981–95.

8. Stone BWG, Dijkstra P, Finley BK, Fitzpatrick R, Foley MM, Hayer M, et al. Life history strategies among soil bacteria—dichotomy for few, continuum for many. ISME J. 2023 Apr;17(4):611–9.

9. Fierer N. Embracing the unknown: Disentangling the complexities of the soil microbiome. Nature Reviews Microbiology. 2017;15(10):579–90.

10. Nelson AR, Narrowe AB, Rhoades CC, Fegel TS, Daly RA, Roth HK, et al. Wildfire-dependent changes in soil microbiome diversity and function. Nature Microbiology. 2022 Aug 25;7(9):1419–30.
